# Methane Reduction Potential of Brown Seaweeds and Their Influence on Nutrient Degradation and Microbiota Composition in a Rumen Simulation Technique

**DOI:** 10.3389/fmicb.2022.889618

**Published:** 2022-06-28

**Authors:** Susanne Künzel, Timur Yergaliyev, Katharina J. Wild, Hanna Philippi, Asta H. Petursdottir, Helga Gunnlaugsdottir, Chris K. Reynolds, David J. Humphries, Amélia Camarinha-Silva, Markus Rodehutscord

**Affiliations:** ^1^Institute of Animal Science, University of Hohenheim, Stuttgart, Germany; ^2^Hohenheim Center for Livestock Microbiome Research, University of Hohenheim, Stuttgart, Germany; ^3^Matís, Reykjavík, Iceland; ^4^Faculty of Food Science and Nutrition, School of Health Sciences, University of Iceland, Reykjavík, Iceland; ^5^School of Agriculture, Policy and Development, University of Reading, Reading, United Kingdom

**Keywords:** seaweed, macro algae, rumen, methane, Rusitec, microbiota, 16S rRNA gene

## Abstract

This study aimed to investigate the effects of two brown Icelandic seaweed samples (*Ascophyllum nodosum* and *Fucus vesiculosus*) on *in vitro* methane production, nutrient degradation, and microbiota composition. A total mixed ration (TMR) was incubated alone as control or together with each seaweed at two inclusion levels (2.5 and 5.0% on a dry matter basis) in a long-term rumen simulation technique (Rusitec) experiment. The incubation period lasted 14 days, with 7 days of adaptation and sampling. The methane concentration of total gas produced was decreased at the 5% inclusion level of *A. nodosum* and *F. vesiculosus* by 8.9 and 3.6%, respectively (*P* < 0.001). The total gas production was reduced by all seaweeds, with a greater reduction for the 5% seaweed inclusion level (*P* < 0.001). Feed nutrient degradation and the production of volatile fatty acids and ammonia in the effluent were also reduced, mostly with a bigger effect for the 5% inclusion level of both seaweeds, indicating a reduced overall fermentation (all *P* ≤ 0.001). Microbiota composition was analyzed by sequencing 16S rRNA amplicons from the rumen content of the donor cows, fermenter liquid and effluent at days 7 and 13, and feed residues at day 13. Relative abundances of the most abundant methanogens varied between the rumen fluid used for the start of incubation and the samples taken at day 7, as well as between days 7 and 13 in both fermenter liquid and effluent (*P* < 0.05). According to the differential abundance analysis with q2-ALDEx2, in effluent and fermenter liquid samples, archaeal and bacterial amplicon sequence variants were separated into two groups (*P* < 0.05). One was more abundant in samples taken from the treatment without seaweed supplementation, while the other one prevailed in seaweed supplemented treatments. This group also showed a dose-dependent response to seaweed inclusion, with a greater number of differentially abundant members between a 5% inclusion level and unsupplemented samples than between a 2.5% inclusion level and TMR. Although supplementation of both seaweeds at a 5% inclusion level decreased methane concentration in the total gas due to the high iodine content in the seaweeds tested, the application of practical feeding should be done with caution.

## Introduction

The rapidly growing global population brings serious challenges to the food industry. While ruminant livestock are vital in sustaining food security by converting inedible plant matter into meat and dairy products, they significantly contribute to global methane (CH_4_) emissions, a potent greenhouse gas (Lassey, 2007). These CH_4_ emissions are mainly related to fermentation processes orchestrated by the rumen microbiome. Diet composition is one of the major factors influencing rumen microbial communities (de Menezes et al., 2011; Henderson et al., 2015) and is therefore linked to CH_4_ production by ruminants.

Feed production does not need arable land, which may help us cope with the increasing demand for animal products. Seaweed could be an alternative animal feed material that has already been used for thousands of years in coastal areas (Evans and Critchley, 2014). Some seaweed species can also affect ruminal CH_4_ production even at a low inclusion level in the feed. Pronounced effects were reported for *Asparagopsis taxiformis* grown in the Pacific Ocean, with CH_4_ formation reduced by up to 99% with seaweed inclusion ≤5% *in vitro* (Kinley et al., 2016; Machado et al., 2018; Roque et al., 2019). Numerous studies have demonstrated that the effect of seaweed supplementation on methanogenesis was associated with a modified rumen microbiome (Molina-Alcaide et al., 2017; Roque et al., 2019; Abbott et al., 2020). In the rumen, hydrogenotrophic methanogens produce CH_4_ by using hydrogen (H_2_) for carbon dioxide (CO_2_) reduction through the Wolfe cycle (Leahy et al., 2010; Thauer, 2012). Accordingly, the composition of archaeal methanogens and abundances of hydrogen-producing bacteria were identified as key factors associated with levels of CH_4_ emissions in ruminants (Tapio et al., 2017). Considering the relevance of H_2_ for CH_4_ production, manipulating H_2_ production or its utilization pathways through diet and microbiome composition is an approach that may provide new insights into the development of CH_4_ mitigation strategies.

Seaweeds are a very heterogeneous group of feeding substances, and their application in animal feeding has been restricted due to a lack of information about species-specific nutritive value. Several factors influence nutrient and bioactive compounds in seaweed, such as species, season, and site of harvesting (Paiva et al., 2018; Britton et al., 2021). To the best of our knowledge, most studies concerning seaweed in ruminant nutrition were conducted with species harvested in the Pacific Ocean. Since there is also a high variation of Atlantic seaweeds, there is a need for information about these seaweeds. The current study aimed to investigate the effect of two Icelandic seaweed samples on *in vitro* gas and methane production, nutrient degradation, and microbiota composition using a continuous long-term rumen simulation technique (Rusitec). Both seaweeds are endemic to Iceland and are also available in large quantities in other European countries. Our hypothesis was that two common and abundant North Atlantic seaweed species could reduce CH_4_ formation to the point where their abundance and ease of access would make them a viable option as a ruminant feed substance. A secondary objective was to investigate changes in microbiota composition over time in the *in vitro* system fed with or without seaweed.

## Materials and Methods

### Treatments

Two samples of seaweed naturally occurring in Iceland were studied for their effect on TMR formulated for cattle in a Rusitec system (Czerkawski and Breckenridge, 1977). The TMR was composed of 20% corn grain, 20% soybean meal, 40% corn silage, and 20% grass silage and served as the control treatment. One seaweed was *Ascophyllum nodosum* (AN), harvested in August 2018, and the other was *Fucus vesiculosus* (FV), harvested in June 2019. Both seaweeds were used at 2.5% (AN2.5 and FV2.5) and 5% (AN5 and FV5) inclusion in the TMR on a dry matter (DM) basis in exchange for TMR. The seaweeds and all ingredients of the TMR were dried and ground to pass a 1 mm screen. The analyzed nutrient composition, bromoform, and total phenolic content (TPC) of the seaweeds and TMR and the calculated nutrient composition of the experimental treatments are shown in Table 1.

**TABLE 1:** Nutrient composition, bromoform, total phenolic content of the used seaweeds, and five experimental diets.

	DM %	OM %	CA %	CP %	ADFom %	aNDFom %	EE %	CHBr_3_ μg/kg	TPC
									Phloroglucinol equivalent g/100 g sample
*A. nodosum*	93.0	70.0	30.0	10.7	18.6	24.1	2.0	8.0	7.9
*F. vesiculosus*	89.5	75.0	25.0	9.3	25.0	19.8	1.8	<0.8	7.4
**Treatments**									
TMR	91.8	94.0	6.0	17.8	14.8	29.6	2.9	n.a.	n.a.
AN2.5^1^	91.8	93.4	6.6	17.6	14.9	29.5	2.8	–	–
AN5^1^	91.8	92.8	7.2	17.4	15.0	29.3	2.8	–	–
FV2.5^1^	91.7	93.6	6.4	17.6	15.0	29.4	2.8	–	–
FV5^1^	91.7	93.1	6.9	17.4	15.3	29.1	2.8	–	–

*AN, Ascophyllum nodosum; FV, Fucus vesiculosus (both with 2.5 or 5% inclusion level); TMR, total mixed ration; DM, dry matter; OM, organic matter; CA, crude ash; CP, crude protein; ADFom, acid detergent fiber on ash free basis; aNDFom, neutral detergent fiber on ash free basis; EE, ether extract; CHBr_3_, bromoform; TPC, total phenolic content; n.a., not analyzed.*

*^1^Values are calculated with respective proportions of TMR and seaweed based on the analyzed values of the ingredients.*

### Rumen Content and Synthetic Saliva

Rumen content was collected from three rumen-fistulated non-lactating Jersey cows before the morning feeding. Animals had free access to water and a diet composed of 33% corn silage, 33% grass silage, 23% grass hay, 10% barley straw, and 1% mineral mixture (on a DM basis). During the daytime, the cows were kept on pasture. Two liters of rumen fluid were taken from each cow into prewarmed thermos flasks, comprised of 1 L pumped from the liquid phase and 1 L squeezed out from the solid phase. Additionally, 200 g of squeezed solid phase from each cow was transferred into prewarmed, isolated containers. Rumen fluid was strained through two layers of cheesecloth and mixed with equal parts from the donor animals. Subsequently, rumen fluid was mixed with a buffer solution (1:1), flushed with CO_2_, and stirred at 39°C until fermenters of the Rusitec were inoculated. The buffer solution was prepared according to the suggested composition of artificial saliva by McDougall (1948) with the addition of ^15^N enriched NH_4_Cl (0.0378 g/L; 104 mg ^15^N/g N) used for the calculation of microbial protein synthesis.

### Rusitec System

**FIGURE 1:**
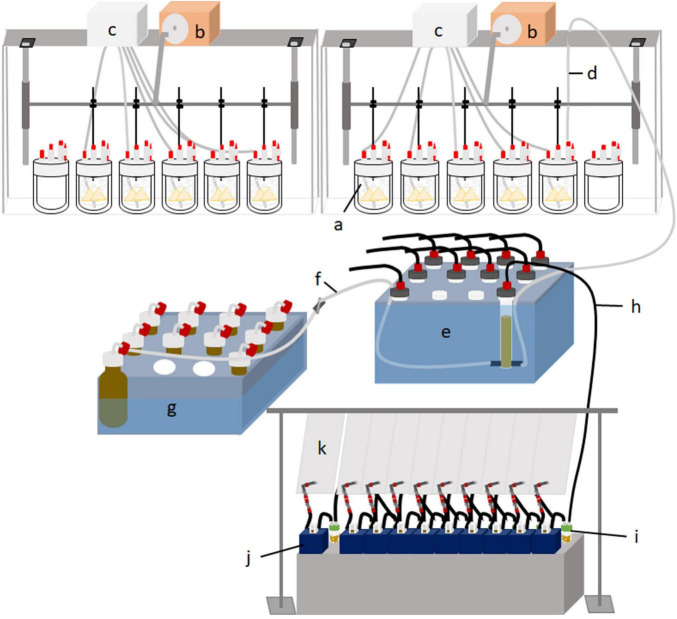
Schematic diagram of the Rusitec set up with the 12 fermenters (a) connected to a lift motor (b), a buffer pump (c), and a gas-tight tube for the effluent (d). The effluent is separated in glass cylinders in a cooling water bath (e) into fluid effluent (f), which is collected in glass bottles in a cooling water bath (g), and gaseous effluent (h). The gaseous effluent is passed through a cold trap with an H_2_S absorber granulate (i), measured with gas counters (j), and sampled for methane analysis in plastic bags (k).

**FIGURE 2:**
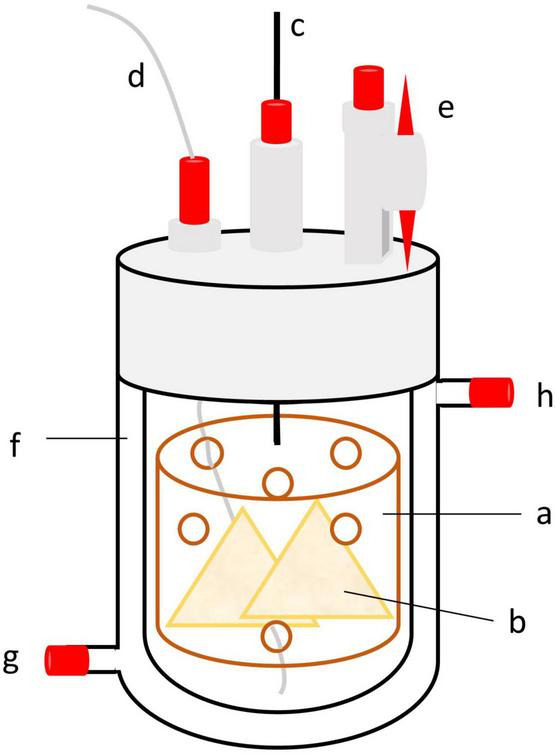
Schematic diagram of a Rusitec fermenter with a feed container (a) containing feed bags (b). The feed container is connected via a bar (c) to a lift motor. Buffer is consistently infused through a tube (d) connected to the buffer pump. The connection to the effluent (e) is closable to make it possible to remove the fermenter. The heating jacket (f) is connected via an inflow (g) and outflow (h) to a circulation thermostat.

Each run lasted 14 days (days 0–13), with 7 days of adaptation, followed by 7 days of sampling. Ten fermenters were arranged side by side, with five fermenters sharing one circulation thermostat (Lauda ECO E 4 S, Lauda-Königshofen, Germany) and one buffer pump (Ismatec IPC ISM 931, Wertheim, Germany) (Figure 1). Each of the five treatments was replicated in two fermenters for two runs each, resulting in four replications per treatment. Fermenters were randomized in each run. The circulation thermostat was used as a block design, and each treatment was used once in each block. Due to technical arrangements, an additional empty fermenter was connected to each circulation thermostat. The glass fermenters were kept at a constant temperature of 39°C with a heating jacket. The buffer pump continuously infused buffer solution into each fermenter at a daily rate of 75% of the fermenter’s capacity (950 ml) to stimulate salivation. Each fermenter contained a feed container with continuous vertical movement ensured by a lift motor to simulate rumen motility (10–12 strokes/min; Figure 2). The fluid effluent was separated from the fermentation gas in a glass cylinder and then collected in 1 L glass bottles. The glass cylinder and effluent bottles were placed in a 4°C tempered water bath. Gaseous effluent was passed through gas counters (BlueVCount, BlueSens gas sensor GmbH, Herten, Germany) to measure gas production via gas-tight tubes and was subsequently collected quantitatively in gas-tight five-layered plastic-aluminum bags (Dr.-Ing. Ritter Apparatebau GmbH & Co., KG, Bochum, Germany). The methane concentration of total gas production was measured using an infrared CH_4_ analyzer from the gas collected in the plastic-aluminum bags (PRONOVA Analysentechnik GmbH & Co., KG, Berlin, Germany).

Feed and solid rumen content were weighed into nylon bags (120 mm × 70 mm) with a 100 μm pore size and closed with cable ties. To start the system, one feed bag containing 15 g of the respective treatment and one feed bag containing 60 g of solid rumen content were put into the container of each fermenter. After 24 h, the bag with rumen content was replaced by another feed bag. Subsequently, each feed bag was removed after 48 h, rinsed with 50 (adaptation period) or 100 ml (sampling period) of buffer solution, squeezed moderately, and replaced by a new bag containing the specific treatment. The resulting liquid was returned to the respective fermenter.

### Sampling and Chemical Analyses

The total gas production and CH_4_ concentration of total gas were measured in 24 h intervals during the sampling period. The temperature, pH, and redox potential of the fermenter fluid were measured daily in the fermenter before the feed bags were changed (SenTix ORP, WTW, Weilheim in Oberbayern, Germany; BlueLine 14 pH IDS, SI Analytics, Mainz, Germany). From days 7 to 13, a sample from the fermenter liquid phase (FL; 30 ml/d) was taken daily and pooled by the fermenter to obtain the fraction of liquid-associated microbes (LAM) by centrifugation according to Boguhn et al. (2006), with minor modifications. In brief, the suspension was centrifuged twice at 2,000 × *g* for 5 min at 4°C. Then, the supernatant was centrifuged three times at 15,000 × *g* for 15 min at 4°C. Afterward, samples were freeze-dried and pulverized with a ball mill (MM 400; Retsch GmbH, Haan, Germany). On day 13, solid-associated microbes (SAM) were separated from the feed residues (FR), as described by Ranilla and Carro (2003). Ammonia nitrogen (NH_3_-N), ^15^N abundance, and volatile fatty acids (VFA) were analyzed in the effluent (E), which was weighed and sampled daily (70 ml/d), pooled by the fermenter, and stored at −20°C until it was centrifuged for 15 min at 24,000 × *g*. The analysis of VFA was performed by vacuum distillation and gas chromatography (Hewlett-Packard 6890; Agilent, Waldbronn, Germany) measurement, as described by Wischer et al. (2013). NH_3_-N was analyzed using Kjeldahl steam distillation with phosphate buffer (Vapodest 50, Gerhardt, Königswinter, Germany). The daily production of VFA and NH_3_-N was calculated by relating the analyzed concentrations to the daily measured amount of E. The removed feed bags were dried for 24 h at 65°C, weighed, and pooled by the fermenter to determine nutrient degradation from days 7 to 12. Feed and FR were analyzed according to official methods in Germany (Verband Deutscher Landwirtschaftlicher Untersuchungs- und Forschungsanstalten, 1976) for DM (method 3.1), crude protein (CP; method 4.1.1), neutral detergent fiber on an ash-free basis (aNDFom; method 6.5.1), and acid detergent fiber on an ash-free basis (ADFom; method 6.5.2). Seaweeds and TMR were additionally analyzed for crude ash (method 8.1) and ether extract (method 5.1.1). Degradation of nutrients was calculated as the difference between the input and output of each fermenter in relation to input and expressed as a percentage.

Bromoform was analyzed in seaweeds according to method 8260B (EPA, 1996) by ALS Global (Miami, FL, United States). For the analysis of TPC, dried seaweeds were extracted in Milli-Q water (1:7 sample:water) for 1 h at room temperature (400 rpm). The TPC was determined on the sample supernatant according to the method by Singleton and Rossi (1965) adapted to the microplate format. For the analysis of heavy metals and minerals in the seaweeds, an ultraWAVE acid digestion system (Milestone Inc., Italy) and an Agilent 7900 quadrupole inductively coupled plasma mass spectrometer (Agilent Technologies, Singapore) were used according to NMKL (2007). Analyzed heavy metals and minerals are shown in Supplementary Table 1. Feed, FR, freeze-dried particle-free E, LAM, SAM, and NH_4_Cl were analyzed for ^15^N abundance using an elemental analyzer (EA, 1108; Carlo Erba Instruments, Biberach, Germany) combined with an isotope mass spectrometer (MS Finnigan MAT; ThermoQuest Italia S.p.A., Milan, Italy). SAM was additionally analyzed for total N.

Microbiota composition was analyzed in rumen solid phase (RSP) and rumen fluid (RF) from the liquid phase of each cow in both experimental runs (*n* = 6), and the mixture of rumen fluid from all cows and buffer solution, treated as RF replicates (*n* = 2) on day 0. On days 7 and 13, 1 ml of FL and E were taken from each fermenter for the analysis of microbiota composition (*n* = 40). Feed residues from both feed bags remaining for 24 and 48 h in the fermenter were sampled on day 13 (*n* = 40). Additionally, the liquid after SAM treatment of both bags was sampled on day 13 (*n* = 20). Samples were stored at −20°C immediately after collection.

### Target Amplicon Sequencing

DNA was extracted with the commercial DNA extraction kit FastDNA™ Spin Kit for soil (MP Biomedicals, LLC, Solon, OH, United States), following the manufacturer’s instructions. DNA was quantified with a NanoDrop 2000 spectrophotometer (Thermo Fisher Scientific, Waltham, MA, United States) and stored at −20°C. Two sequencing libraries were prepared as previously described to assess bacterial (V1–V2 region; Kaewtapee et al., 2017) and archaeal communities (Arch349 and Arch806 primers; Lee et al., 2012; Deusch et al., 2017). In brief, targeted regions of the 16S rRNA gene were amplified by a first polymerase chain reaction (PCR) using forward primers with 6-nt barcodes and a 2-nt linker attached and both primers were complementary to the Illumina adapters. Then, 1 μl of the resulting product was used as a template for a second PCR, this time using a reverse primer containing a specific sequence to multiplex and index primers. Obtained amplicons were checked by agarose gel electrophoresis and normalized using the SequalPrep Normalization Kit (Invitrogen Inc., Carlsbad, CA, United States). Sequencing was performed on the 250 bp paired-end Illumina NovaSeq 6000 platform.

### Calculations

The estimated microbial protein synthesis (EMPS) was calculated in accordance with Boguhn et al. (2006) and Hildebrand et al. (2011). In brief, the daily outflow of microbial N from a fermenter (N_m_; mg/d) was calculated as the sum of N originating from the LAM in the effluent (N_LAM–E_; mg/d) and SAM (N_SAM_; mg/d) fractions:


(1) $$N_{m}=N_{LAM-E}+N_{SAM}$$

Therefore, N_LAM–E_ was calculated with the sum of the daily input of ^15^N via buffer solution and feed (^15^N_in_; μg/d), the sum of the daily output of ^15^N via FR and NH_3_-N (^15^N_out_; μg/d), and the analyzed abundance of ^15^N in N_LAM–E_ (^15^N_LAM–E_; μg/mg N):


(2) NLAM-E=Nin15-Nout15NLAM-E15

The N_SAM_ was calculated using the amount of N in FR (N_FR_; mg/day), the analyzed abundances of ^15^N in the FR N (^15^N_FR_; μg/mg N), the feed N (^15^N_feed_; μg/mg N), SAM N (^15^N_SAM_, μg/mg N), and the assumed natural abundance of ^15^N in unlabeled N_SAM_ (3.66303 μg/mg):


(3) NSAM=NFR×NFR15-NFeed15NSAM15-3.66303

Microbial CP (CP_m_; mg/d) was calculated as N_m_ (Eq. 1) multiplied by 6.25:


(4) $$CP_{m}=N_{m}\times 6.25$$

In the next step, EMPS [g/kg degraded organic matter (OM)] was determined using the CP_m_ (mg/d), the degraded organic matter (OM_deg_; mg/d), and the amount of OM originating from SAM in the FR (OM_SAM_; mg/d):


(5) $$EMPS=\frac{CP_{m}}{OM_{deg}+OM_{SAM}}$$

Where OM_SAM_ was calculated as described by Boguhn et al. (2006) with the amount of N_SAM_ (Eq. 3), the analyzed N concentration in SAM (N_%SAM_; %), the concentration of ash in SAM [12%, Boguhn et al. (2006)], and the proportion of DM in the isolated SAM fraction [0.93, Boguhn et al. (2006)]:


(6) $$OM_{SAM}=\frac{N_{SAM}}{N_{\%SAM}}\times \left(100-12\right)-0.93$$

### Statistical Analyses and 16S Bioinformatics

Statistical analysis of gas data, nutrient degradation, NH_3_-N, VFA, and EMPS was done with a one-way ANOVA in SAS 9.4 using the mixed procedure. The treatment was the fixed effect, and the run, circulation thermostat, fermenter, and day were used as random effects. When treatment differences were identified, multiple *t*-tests (Fisher’s LSD test) were used to distinguish between treatments. The residuals were checked graphically for the normal distribution and homogeneity of variance.

Fastq files were demultiplexed with Sabre^1^ and processed by Qiime2 (v.2021.2/8; Bolyen et al., 2019). Primers were removed by the q2-cutadapt plugin (Martin, 2011). Reads were quality filtered, error corrected, dereplicated, and merged by the q2-dada2 plugin (Callahan et al., 2016). Taxonomy assignment of produced amplicon sequence variants (ASVs) was performed using VSEARCH-based consensus (Rognes et al., 2016) and pre-fitted sklearn-based classifiers (Pedregosa et al., 2011) against the Silva SSU-rRNA database (v.138.1, 16S 99%; Quast et al., 2013), formatted by RESCRIPt (Robeson et al., 2021). Reads from organelles and unassigned sequences were removed from the analysis. A phylogenetic tree was constructed with the q2-phylogeny plugin, implementing MAFFT 7.3 (Katoh and Standley, 2013) and FastTree 2.1 (Price et al., 2010). For diversity assessment, ASV tables were rarefied to 3580 (archaea) and 5856 (bacteria) sampling depths. Alpha diversity was estimated by Faith’s phylogenetic diversity (Faith, 1992) and Shannon’s entropy (Shannon, 1948) indices. For beta diversity, Bray–Curtis (Bray and Curtis, 1957) and Jaccard (Jaccard, 1912) distances were employed. Ordination of the beta-diversity distances was implemented with a principal-coordinate analysis (PCoA; Halko et al., 2011). Differences in diversity metrics were tested with the Kruskal–Wallis *H*-test (Kruskal and Wallis, 1952; alpha diversity and relative abundances), followed by Dunn’s pairwise test (Dunn, 1964) and a PERMANOVA test (Anderson, 2001; beta diversity) with 999 permutations. The obtained *P*-values were corrected for multiple comparisons using the Benjamini–Hochberg procedure (Benjamini and Hochberg, 1995).

For correlation analysis, ASV tables were filtered to remove genera with less than 1% relative abundances. Absolute abundances of the remaining genera were correlated using “Sparse Cooccurrence Network Investigation for Compositional Data” or the SCNIC tool (Shaffer et al., 2020). Genera were correlated using the SparCC method (Friedman and Alm, 2012) and with numerical metadata using the Spearman correlation (Dodge, 2010). All *P*-values were considered to be significant if ≤0.05 (except for q2-ALDEx2 output) and correlation coefficients (r) if their absolute values were ≥0.3.

The q2-ALDEx2 differential abundance plugin was used to test the effect of seaweed supplementation on ASVs raw counts (relative abundance ≥1%) with a significance threshold of Wilcoxon test 0.1 (Fernandes et al., 2013, 2014). Sequences are available at the European Nucleotide Archive (ENA) under accession number PRJEB50942 and bioinformatics^2^.

## Results

### Gas Production, Nutrient Degradation, and Microbial Protein Synthesis

The total gas production was decreased by both seaweeds compared to TMR alone, with a greater effect for the 5% seaweed inclusion level (*P* < 0.001, Table 2). No significant difference between AN and FV was detected for total gas production. For the CH_4_ concentration of total gas and the methane production per g of degraded OM, only the 5% inclusion level of the two seaweeds showed a difference compared to TMR alone (*P* < 0.001 and 0.017, respectively). The reduction was bigger for AN5 (8.9 and 16.9% reduction for CH_4_ concentration and CH_4_/g degraded OM) compared to FV5 (3.6 and 11.2% reduction for CH_4_ concentration and CH_4_/g degraded OM).

**TABLE 2:** Total gas production and methane concentration of the produced gas (days 7–13) and methane production per g of degraded organic matter (OM; days 7–12) of the five treatments.

	Total gas (mL/d)	CH_4_ (% of total gas)	CH_4_/degraded OM (mL/g)
TMR	1386^a^	16.9^a^	36.7^a^
AN2.5	1238^b^	16.6^ab^	35.1^ab^
AN5	1114^c^	15.4^c^	30.5^c^
FV2.5	1177^bc^	16.6^ab^	35.9^ab^
FV5	1114^c^	16.3^b^	32.6^bc^
*Pooled SEM*	*35.9*	*2.01*	*4.66*
*P*	<0.001	<0.001	0.017

*AN, Ascophyllum nodosum; FV, Fucus vesiculosus (both with 2.5 or 5% inclusion level); TMR, total mixed ration.*

*^a–c^Means within a column not showing a common superscript differ (P < 0.05).*

Seaweed supplementation also decreased the degradation of all analyzed nutrients in the feed bags (*P* < 0.001, Table 3). Only for ADFom, AN2.5 showed no significant difference to TMR alone. In all other cases, nutrient degradation was significantly lower with seaweed supplementation. The 5% inclusion level caused at least a numerically greater reduction than the 2.5% inclusion. The highest decrease compared to TMR alone was observed for CP, with a minimum reduction of 8.9% percentage points. The two seaweed products only differed at the 5% inclusion level for DM and OM degradation, where FV showed a greater decrease than AN. The EMPS was decreased by both seaweeds, with a greater effect of the 5% inclusion level (*P* < 0.001, Table 3). No significant difference was detected between the inclusion of AN and FV.

**TABLE 3:** Nutrient degradation in the feed bags of the five treatments (days 7–12) and estimated microbial protein synthesis (days 7–13).

	Degraded					EMPS
	DM	OM	CP	ADFom	aNDFom	
		
	%					mg/g degraded OM
TMR	44.8^a^	44.3^a^	39.5^a^	14.0^a^	24.7^a^	120^a^
AN2.5	41.2^b^	40.7^b^	30.6^b^	11.5^ab^	21.8^b^	109^b^
AN5	39.5^c^	39.2^b^	25.7^c^	8.5^c^	18.7^c^	100^c^
FV2.5	40.4^bc^	40.0^b^	30.4^b^	11.1^b^	20.5^bc^	110^b^
FV5	37.7^d^	37.3^c^	25.1^c^	9.7^bc^	18.9^c^	100^c^
*Pooled SEM*	*0.66*	*0.73*	*0.82*	*0.99*	*0.89*	*10.8*
*P*	<0.001	<0.001	<0.001	0.001	<0.001	<0.001

*AN, Ascophyllum nodosum; FV, Fucus vesiculosus (both with 2.5 or 5% inclusion level); TMR, total mixed ration; DM, dry matter; OM, organic matter; CP, crude protein; ADFom, acid detergent fiber on ash free basis; aNDFom, neutral detergent fiber on ash free basis; EMPS, estimated microbial protein synthesis.*

*^a–d^Means within a column not showing a common superscript differ (P < 0.05).*

### Fermentation Characteristics

The temperature (*P* = 0.576; 38.6°C) and redox potential measured in FL (−241 mV; *P* = 0.062) did not differ among the treatments. The pH in the fermenter was different between the TMR alone (pH = 6.82) and the seaweed supplemented treatments (all pH = 6.85; *P* = 0.004). At both supplementation levels, seaweed decreased analyzed NH_3_-N and VFA production compared to TMR alone, except for propionate production in FV2.5 (Table 4; *P* ≤ 0.001). For isovalerate and valerate, the 5% inclusion level of both seaweeds showed a less pronounced reduction than the 2.5% inclusion level. For butyrate, this was only the case for seaweed AN and acetate only for seaweed FV. For NH_3_-N and the other VFA, the decrease was equal to or greater for the 5% inclusion level than the 2.5%.

**TABLE 4:** NH_3_-N and VFA production analyzed in the effluent of the five treatments (days 7–13).

	NH_3_-N	Acetate	Propionate	Isobutyrate	Butyrate	Isovalerate	Valerate	VFA_total_	C2:C3
	mmol/d	mmol/d	mmol/d	mmol/d	mmol/d	mmol/d	mmol/d	mmol/d	
TMR	5.40^a^	19.7^a^	7.00^a^	0.40^a^	7.85^a^	1.40^a^	3.08^a^	39.3^a^	2.86^a^
AN2.5	3.39^b^	17.3^b^	6.56^cd^	0.28^b^	5.27^c^	0.82^c^	2.29^d^	32.5^bc^	2.63^b^
AN5	2.73^d^	16.4^c^	6.57^d^	0.26^c^	5.86^b^	0.89^b^	2.97^b^	32.9^b^	2.47^bc^
FV2.5	3.42^b^	16.7^c^	6.83^ab^	0.27^c^	5.35^c^	0.77^d^	2.45^c^	32.4^bc^	2.42^cd^
FV5	2.84^c^	15.4^d^	6.78^bc^	0.26^c^	5.39^c^	0.89^b^	2.98^b^	31.7^c^	2.25^d^
*Pooled SEM*	*0.140*	*0.71*	*0.335*	*0.008*	*0.469*	*0.069*	*0.177*	*0.83*	*0.062*
*P*	<0.001	<0.001	<0.001	<0.001	<0.001	<0.001	<0.001	<0.001	<0.001

*AN, Ascophyllum nodosum; FV, Fucus vesiculosus (both with 2.5 or 5% inclusion level); TMR, total mixed ration.*

*^a–d^Means within a column not showing a common superscript differ (P < 0.05).*

### Alpha and Beta Diversity

**FIGURE 3:**
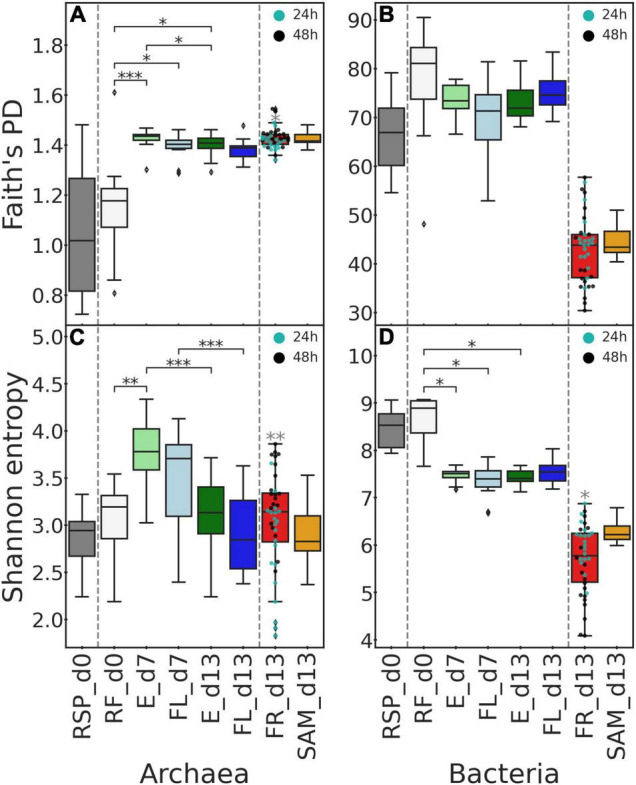
Alpha diversity indices by sample type and sampling day. Faith’s phylogenetic diversity **(A,B)** and Shannon entropy index **(C,D)** are plotted for archaea **(A,C)** and bacteria **(B,D)**. Boxplots visualize the alpha diversity metric distribution across all samples inside a given group. Gray dashed lines subdivide boxes into groups, where pairwise comparisons were performed. Significant after Benjamini–Hochberg correction, *P*-values of Dunn’s pairwise test are denoted by black asterisks. For FR samples, additional (dots) were plotted, indicating samples taken from bags with 24 and 48 h incubation time. Gray asterisks are plotted above FR samples if the Kruskal–Wallis test indicated a significant difference between samples with different incubation times. Significant *P*-values denoted as: *P ≤ 0.05, ^**^P ≤ 0.01, and ^***^P ≤ 0.001.

After sequencing and demultiplexing, a total of 4,843,225 and 5,851,973 read pairs were obtained for archaeal and bacterial datasets, respectively. Quality filtering, denoising, merging of paired reads, and chimera removal resulted in 2,648,201 archaeal and 4,237,733 bacterial ASVs. Archaeal Faith’s PD and Shannon indices were approximately equal in RF and RSP at the start of the experiment (Figures 3A,C and Supplementary Table 2) and failed to reject the null hypothesis when tested against each other (Faith’s PD *P* = 0.881, Shannon *P* = 0.531). Archaeal phylogenetic diversity was greater in the Rusitec samples compared to the inoculum; meanwhile, Shannon entropy fluctuated in the system among sampling days. Compared to RF, Faith’s PD was greater in E and FL at day 7 (*P* = 0.001 and 0.044) and in E at day 13 (*P* = 0.026). E phylogenetic diversity at day 13 decreased compared to day 7 (*P* = 0.048). Shannon entropy at day 7 was greater in E than RF (*P* = 0.006). The entropy of E and FL sample types was reduced from days 7 to 13 (*P* = 0.001). At day 13, both metrics demonstrated no differences between FR and SAM samples (all *P* > 0.05) but were greater in FR samples from bags with a 48-h incubation period compared to 24 h (Faith’s PD *P* = 0.028, Shannon *P* = 0.001). In the bacterial dataset, the alpha diversity of samples at day 0 (RF and RSP) was also at the same level (all *P* > 0.05) (Figures 3B,D and Supplementary Table 2). Unlike archaea, bacterial communities demonstrated no changes in E and FL samples compared by sampling days and the initial RF at day 0 (all *P* > 0.05). Meanwhile, the Shannon index decreased in E and FL at day 7 (*P* = 0.029 and 0.017) and in E at day 13 (*P* = 0.019). Both tested alpha diversity metrics of FR and SAM revealed no differences between these sample types (all *P* > 0.05) but were lower for both of them than in all samples from days 0 to 7 and E and FL at day 13 (all *P* < 0.05). When testing FR samples based on incubation time, no differences were observed for phylogenetic diversity, but Shannon entropy was lower in 48-h samples (*P* = 0.026).

In archaea, seaweed additives affected the Shannon entropy of E at days 7 and 13 and FL at day 13 (Supplementary Figure 1). At both days 7 and 13, diets AN5, FV2.5, and FV5 resulted in lower entropy than the TMR alone (all *P* < 0.05). In FL, the Shannon index of FV2.5 and FV5 samples was also lower than that of TMR (all *P* < 0.05). Among bacteria, no significant changes between treatments were observed according to both alpha diversity metrics (all *P* > 0.05).

**FIGURE 4:**
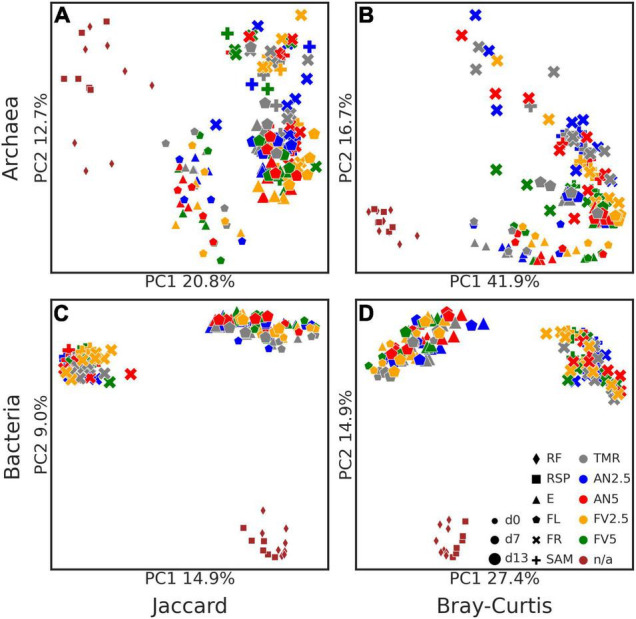
Principal coordinates analysis (PCoA) plots of beta diversity metrics. Jaccard **(A,C)** and Bray–Curtis **(B,D)** distances are plotted for archaea **(A,B)** and bacteria **(C,D)**. Points are sized by the sampling day (days 0–13), shaped by the sample type, and colored by treatments [as stated in the legend on subplot **(D)**]. Label “n/a” in the legend means no treatment was applied.

Concerning beta diversity, PCoA analysis (Figure 4) was performed on the ASVs, using Jaccard and Bray–Curtis distances. Archaeal and bacterial samples clustered into three big groups (RF and RSP from day 0 as one group, E and FL samples from days 7 to 13 as another one, and FR and SAM from day 13 as the third), with clearer clusters in the archaeal dataset. PERMANOVA analysis revealed that microbial composition was affected by the sample type (all *P* < 0.05), sampling day (all *P* < 0.05), and between FR samples incubated for 24 and 48 h (all *P* < 0.01), except for archaeal Jaccard distances.

Seaweed inclusion affected archaeal beta diversity according to both Jaccard and Bray–Curtis distances of E samples at days 7 and 13 and FL at day 13 (all *P* < 0.05). Additionally, Bray–Curtis distances were also affected in FR and SAM samples (all *P* < 0.05). Pairwise tests with correction for multiple comparisons found differences in Bray–Curtis distances of FR samples between the FV5 diet and TMR alone and AN2.5 (all *P* = 0.025). No effect of seaweed on bacterial beta diversity was detected (all *P* > 0.05).

### Archaeal and Bacterial Taxonomy Composition

**FIGURE 5:**
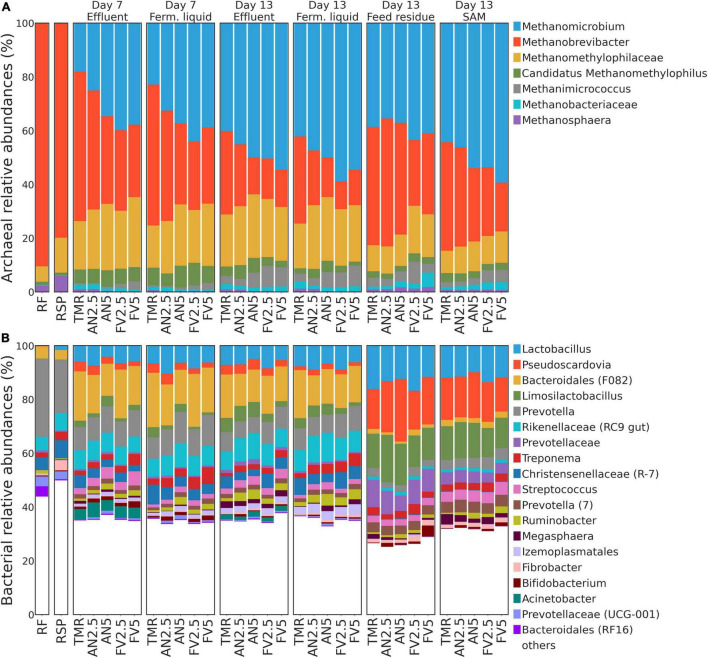
Stacked taxonomy bar plots. Relative abundances of **(A)** archaeal and **(B)** bacterial genera are plotted for each sample type and sampling day by treatments (only genera with relative abundance ≥3.5% among at least one column were included). If certain features were not annotated to genus level, the last available taxonomy unit was shown. Minor Silva annotation identifiers, such as clades, taxonomic groups, clones, and others not included in commonly used nomenclature, are indicated in the round brackets.

Most of the archaeal sequences (≈ 95% of the reads) were assigned to *Methanomicrobium, Methanobrevibacter*, unclassified to genus level members of the Methanomethylophilaceae family, *Candidatus Methanomethylophilus*, and *Methanimicrococcus* (Figure 5A). *Methanobrevibacter* was the most abundant genus in RSP and RF samples, with a gradual shift toward *Methanomicrobium* dominance in E and FL. Considering all samples, regardless of the treatment by sample type and day (Supplementary Table 3), relative abundances of *Methanomicrobium* were barely detectable in the samples that served as inoculum RF and RSP but increased up to the dominance in samples from the Rusitec (all *P* < 0.05). On the contrary, *Methanobrevibacter* ratios mostly decreased in the Rusitec samples (all *P <* 0.05, except for E at day 7 compared to RF: *P* = 0.065). Moreover, *Methanomicrobium* relative abundances in E and FL were also greater at day 13 than at day 7 (*P* = 0.01 and 0.001); meanwhile, by the same comparison, the presence of *Methanobrevibacter* at day 13 was lower for E (*P* = 0.03). Except for SAM, the proportions of unclassified *Methanomethylophilaceae* in RF were lower than in Rusitec sample types (all *P* < 0.05). Methanomicrobium in FL at day 13 (*P* = 0.023), unidentified Methanomethylophilaceae in E at day 7 (*P* = 0.029), Methanimicrococcus in E at day 7 (*P* = 0.023), and FL at day 13 (*P* = 0.008) had higher relative abundances in FV5 samples than TMR alone (Supplementary Table 4). At day 13, *Methanimicrococcus* was also more abundant in FV2.5 samples from SAM (*P* = 0.043).

On average, among bacteria, *Lactobacillus* showed the highest abundance across all samples, followed by *Pseudoscardovia*, unclassified Bacteroidales, marked by Silva database as F082 (further F082), *Limosilactobacillus*, and *Prevotella* (Figure 5B). The high abundance of *Lactobacillus* was mostly observed in FR and SAM samples. In E and FL samples, F082 dominated the community. *Prevotella* accounted for the highest abundance in the inoculum (day 0) but was less abundant in E, FL, FR, and SAM (all *P* < 0.05) compared to RF, except for E at day 7. Compared with sample types and sampling days (Supplementary Table 3), relative abundances of *Lactobacillus, Pseudoscardovia*, and F082 were about the same in E and FL at both days 7 and 13 and increased their proportions compared to cow samples (all *P* < 0.05, except for *Pseudoscardovia* at day 13) after incubation in the Rusitec. Notably, *Lactobacillus* and *Pseudoscardovia* were poorly represented in RF and RSP. Treatment AN5 decreased relative abundances of F082 in E samples on day 7 (*P* = 0.025) and FR on day 13 (*P* = 0.005) compared to TMR alone. Meanwhile, *Ruminobacter* proportions in E (*P* = 0.044) and SAM samples (*P* = 0.05) increased at day 13. FV5 also increased relative abundances of *Ruminobacter* in FL samples at day 13 (*P* = 0.022) (Supplementary Table 4).

### Effect of Seaweed Additives on the Microbial Amplicon Sequence Variant Abundances

**FIGURE 6:**
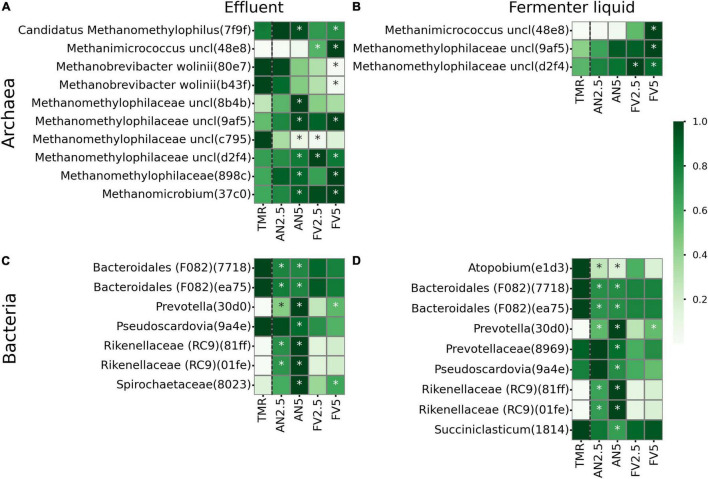
Relative abundances of differentially abundant features according to ALDEx2. Relative abundances are normalized by rows. Archaeal **(A,B)** and bacterial **(C,D)** features from effluent **(A,C)** and fermenter liquid **(B,D)** sample types. All seaweed additives were compared with TMR. ALDEx2 significant abundances are marked by asterisks. All supplements were compared to TMR alone and separated by black dashed lines.

In E and FL sample types, 10 archaeal and 10 bacterial ASVs were differentially abundant in seaweed-supplemented diets compared to TMR alone (Figure 6). In E, archaeal ASV with the first four characters in corresponding id digest 7f9f (further indicated in round brackets), assigned to *Candidatus Methanomethylophilus*, was more abundant in treatments AN5 (*P* = 0.054) and FV5 (*P* = 0.049). *Methanobrevibacter wolinii* ASVs (80e7, b43f) were less abundant in FV5-supplemented treatments (*P* = 0.013, 0.01); meanwhile, *Methanomicrococcus* (48e8) abundances increased (*P* = 0.002). Unclassified *Methanomethylophilaceae* ASVs (d2f4, 8b4b, 9af5, 898c, and 37c0) were mostly associated with seaweed-supplemented samples, with one ASV (c795) more abundant in TMR alone and decreased when the FV2.5 (Wilcoxon *P* = 0.008) was added. In FL, *Methanomicrococcus* (48e8) (*P* = 0.005) and *Methanomethylophilaceae* (9af5, d2f4) (*P* = 0.067, 0.069) were more abundant in FV5 diet. *Methanomethylophilaceae* (d2f4) also increased when FV2.5 seaweed was added (*P* = 0.052).

Among bacteria of E and FL sample types, ASVs (7718, ea75), assigned to the Bacteroidales F082 group, *Pseudoscardovia* (9a4e), *Atopobium* (e1d3), *Prevotellaceae* (8969), and *Succiniclasticum* (1814) were mostly associated with TMR alone. However, *Prevotellaceae* (8969) (*P* = 0.083) and *Pseudoscardovia* (9a4e) (*P* = 0.085) were more abundant in FL samples of AN5. *Prevotella* (30d0) was more abundant in AN2.5, AN5, and FV5 samples (*P* = 0.002, 0.001, and 0.011 in FL and 0.002, 0.003, and 0.005 in E). *Rikenellaceae* RC9 gut group (81ff, 01fe) also increased in E and FL sample types when AN seaweed was added (*P* in the range of 0.002–0.003). Finally, in E unclassified *Spirochaetaceae* (8023) was more abundant in AN5 (*P* = 0.004) and FV5 (*P* = 0.04) supplemented diets.

### Correlations

A correlation analysis was performed to investigate potential relationships between microbiota genera, total gas, CH_4_ concentration, and VFA production (Supplementary Tables 5–7). At the genus level, correlations of microbial abundances with total gas production, CH_4_ concentration, and VFA were performed for E, FL, and FR sample types (Supplementary Figure 2), with only significant correlations plotted (absolute value of Spearman *r* ≥ 0.3 and *P* ≤ 0.05). The largest number of genera that negatively correlated with the above-mentioned traits was detected in E samples; meanwhile, in FL and FR, positive correlations prevailed.

## Discussion

### Seaweed Effects

A significant reduction of CH_4_ concentration in the total gas was found to be caused by the supplementation of both seaweed species at the 5% inclusion level. However, the effect was not as high as previously reported for *A. taxiformis* (Kinley et al., 2016; Machado et al., 2018; Roque et al., 2019). Additionally, the lower CH_4_ concentration in total gas was accompanied by a reduced rate of overall fermentation. The microbiota produced not only less methane but also fewer metabolites relevant to animals. This was indicated by the decreased total gas production, VFA, NH_3_-N, and nutrient degradation. Reduced fermentation and nutrient degradation would generally mean a diminished amount of nutrients and energy available for the host animal. However, in the case of CP, a reduced degradation could benefit the animal because the ruminal undegradable protein could be used as a bypass protein like it was already shown for other seaweed species (Tayyab et al., 2016). The reduced CP degradation may have resulted from the formation of complexes between the phlorotannins contained in both seaweeds and other proteins (Belanche et al., 2016) and resulted in a lower EMPS, but more research is needed to confirm this.

The CH_4_ reduction effect of *A. taxiformis* is attributed to the presence of bromoform (Machado et al., 2016), which is known to be a carcinogen (National Toxicology Program, 1989). The suggested modes of action in the brown seaweeds AN and FV used in the present study were likely related to phlorotannins as the bromoform content [8.0 (AN) and <0.8 μg/kg DM (FV)] was negligibly lower compared to the one analyzed by Machado et al. (2016) for *A. taxiformis* (1,723,000 μg/kg DM). The TPC, an indicator of phlorotannin content, was very similar in both seaweeds (7.9 vs. 7.4 phloroglucinol equivalent g/100 g sample). The greater CH_4_ reduction with the supplementation of AN compared to FV could be explained by either the slightly greater TPC or the greater bromoform content in this sample, even if it is still very low. However, both species showed the same CH_4_ concentration of total gas at the low seaweed inclusion level. Both bromoform and terrestrial tannins influence methanogens (Cieslak et al., 2014; Machado et al., 2018). Additionally, it has been shown that the composition of archaeal methanogens is associated with CH_4_ production (Tapio et al., 2017). Consistent with this finding, our study showed that the decline in methane concentration of total gas in seaweed supplemented samples was accompanied by the separation of archaeal and bacterial ASVs in a dose-dependent manner, indirectly supporting the importance of methanogen composition. Interestingly, AN5 seaweed supplementation resulted in more differentiation of microbial ASVs abundances and a stronger reduction of CH_4_ concentration when compared to TMR than FV5.

The treatment showing the highest CH_4_ reduction in this study, AN5, also contained the highest iodine concentration (Supplementary Table 1). The iodine content of AN was more than 10 times greater than that of FV. Therefore, the application of practical feeding should be done with caution. Iodine toxicity in dairy cows is reported by the National Research Council (U.S.) (2001) for a concentration of 5 mg iodine/kg dietary DM. The European Food Safety Authority recommends a maximum iodine content of 2 mg/kg of complete feed (EFSA, 2013). In the current experiment, the iodine concentration was 35 mg/kg DM for treatment AN2.5 and 2.75 mg/kg DM for treatment FV2.5. Additionally, it was shown that the iodine concentration in milk follows a dose-response relationship with iodine intake (Antaya et al., 2015; Newton et al., 2021). Humans, especially children, are even more sensitive to iodine poisoning than ruminants (Zimmermann et al., 2005). Therefore, the seaweed inclusion levels used in the present study do not apply to practical ruminant feeding, except for FV2.5. Seaweed supplementation at an exceptional iodine inclusion level would therefore not significantly reduce methane compared to TMR.

### Methanogenesis

In our study, *Prevotella*, unclassified members of *Prevotellaceae* and *Clostridia* negatively correlated with CH_4_ concentration (Supplementary Table 6) and archaeal methanogens (Supplementary Table 7). *Prevotella* spp. and other members of the *Prevotellaceae* family are known for their ability to consume H_2_ and produce propionate (Strobel, 1992; Mitsumori et al., 2012; Denman et al., 2015), which in turn is negatively associated with CH_4_ formation and able to act as an alternative hydrogen sink (Ungerfeld, 2015). Inverse associations of *Prevotella* spp. with CH_4_ concentration were previously reported (Mitsumori et al., 2012; Aguilar-Marin et al., 2020). Positive correlations with CH_4_ concentration were observed for *Fibrobacter, Lactobacillus, Pseudoramibacter, Olsenella, Shuttleworthia*, and unclassified *Oscillospiraceae* (NK4A214). *Fibrobacter*, a *fibrolytic* bacteria that produce formate, can also be utilized by methanogens as a substrate for CH_4_ production (Rychlik and May, 2000). Positive correlations of the *Fibrobacter* genus with CH_4_ formation were previously reported (Wang et al., 2016). The addition of some *Lactobacillus* and *Limosilactobacillus* spp. (*L. mucosae*) in an *in vitro* rumen fermentation technique led to the rise of CH_4_ production (Soriano et al., 2014). *Pseudoramibacter* can utilize carbohydrates as an energy source (Deusch et al., 2017), producing VFA during fermentation, including butyrate, acetate, formate, and hydrogen (Palakawong Na Ayudthaya et al., 2018), and increasing methane production by methanogens. *Olsenella* contains genes encoding choline trimethylamine lyase (Kelly et al., 2019) and is involved in CH_4_ production (Broad and Dawson, 1976; Neill et al., 1978). *Shuttleworthia* is positively correlated with CH_4_ formation in dairy heifers (Cunha et al., 2019) and produces acetate and butyrate through glucose fermentation (Downes et al., 2002). *Oscillospiraceae* members were linked with acetate production (Tanca et al., 2017) and, in our study, correlated with acetate, the A:P ratio, and CH_4_ concentration. In general, those correlations are consistent with previous studies. It was shown that the addition of AN to an *in vitro* fermentation system led to a decrease in the growth of *Fibrobacter succinogenes* (positively correlated with CH_4_ concentration) and an increase in *Prevotella bryantii* (negatively correlated with CH_4_ concentration) (Wang et al., 2009). As it was reviewed by Abbott et al. (2020), it is possible that seaweeds modulate the growth of cellulolytic rumen bacteria by altering polysaccharide availability (Lee et al., 2019).

### Adaptation of Microbiota Composition

Although we allowed rumen microbiota to adapt for 7 days in the Rusitec prior to sampling, archaeal community distribution demonstrated a shift in the dominant genera at the end of the adaptation period and revealed a variation between the samplings at days 7 and 13. Partially, it can be explained by lower metabolite digestibility in the Rusitec than *in vivo* conditions and lack of or low protozoa counts (Martínez et al., 2010; Hristov et al., 2012). Relative abundances of *Methanobrevibacter*, which is the most abundant methanogen genus in cow samples, continuously decreased in the Rusitec. This tendency was somehow more profound in seaweed-treated samples, although no statistically significant differences with TMR were detected. In the Rusitec samples, dominance gradually shifted from *Methanobrevibacter* to *Methanomicrobium* by day 13. Although it has been shown that the Rusitec provides a stable condition for the methanogens (Lengowski et al., 2016), additional studies with longer *in vitro* incubation periods may be performed to investigate *Methanobrevibacter/Methanomicrobium* ratio dynamics in such systems since abundances of *Methanobrevibacter* relative to other archaea may affect CH_4_ production (Cunha et al., 2019). When the total gas production and methane concentration are additionally statistically analyzed not only by treatment but also by sampling day and the interaction between treatment and day, it becomes evident that both traits increased during the experimental period (*P* = 0.005 and <0.001, respectively; Supplementary Table 8). This coincides with changes in the proportion of the most abundant methanogens in the system (*Methanobrevibacter* abundances decreased, while *Methanomicrobium* increased) between days 7 and 13 and indicates that adaptation of the microbes to the system was not finalized after day 7. Previous studies have shown a decrease in the protozoa population, also known as a methane producer, in Rusitec fermenters over time (Ziemer et al., 2000; Martínez et al., 2010; Morgavi et al., 2012). Protozoa were not analyzed in the current study, but this potential shift could also have led to an altered archaeal community distribution.

### Conclusion

The North Atlantic seaweed species used herein can modify the microbiota in the rumen toward reduced methane production. However, methane reduction was not great, and the seaweed inclusion in the ration caused reduced *in vitro* fermentation overall. Therefore, the tested seaweed species are not a viable option to be used as feed for ruminants to mitigate methane production.

## Data Availability Statement

The data presented in the study are deposited in the European Nucleotide Archive (ENA) repository, accession number PRJEB50942 (https://www.ebi.ac.uk/ena/browser/view/PRJEB50942).

## Ethics Statement

The animal study was reviewed and approved by the Regierungspräsidium Stuttgart, Germany.

## Author Contributions

SK, KW, MR, AC-S, HG, and AP conceived and designed the experiment. SK, KW, and HP performed the experiments. SK, KW, HP, and MR performed the *in vitro* data analysis. TY and AC-S performed the microbial data analysis. AP and HG carried out chemical data analysis. SK and TY drafted the manuscript. All authors revised and approved the manuscript revisions.

## Conflict of Interest

The authors declare that the research was conducted in the absence of any commercial or financial relationships that could be construed as a potential conflict of interest.

## Publisher’s Note

All claims expressed in this article are solely those of the authors and do not necessarily represent those of their affiliated organizations, or those of the publisher, the editors and the reviewers. Any product that may be evaluated in this article, or claim that may be made by its manufacturer, is not guaranteed or endorsed by the publisher.

## Footnotes

1
https://github.com/najoshi/sabre

2
https://github.com/timyerg/Kunzel_rstc_2019

## Supplementary Material

Supplementary Figure 1 Alpha diversity indices by treatment, sample-type and day of sampling. Faith’s phylogenetic diversity and Shannon entropy index are plotted for archaea and bacteria. Each subplots represents one of alpha diversity metrics at a certain day of sampling. Boxplots visualize alpha diversity metrics distribution across all treatments inside of a certain group. Kruskal–Wallis *H*-test was performed to test the differences between treatments alpha diversity and corresponding *P*-values are plotted on the top of subplots. Significant *P*-values denoted as ‘*’ if *P* ≤ 0.05.

Supplementary Figure 2 SCNIC based correlations of microbial relative abundances among effluent, fermenter liquid and feed residue sample types with total gas production, methane as a percentage of total gas [CH4(%)], ammonium nitrogen in effluent (NH_3_-N), VFA production and acetate to propionate ratio (A:P ratio). Only correlation coeffitients (*r*) with absolute values ≥0.3 and adjusted *P*-values ≤ 0.05 are plotted. Positive correlations are colored in read and negative in blue.

## Abbreviations

ADFom: acid detergent fiber on ash-free basis
AN: *Ascophyllum nodosum*
aNDFom: Neutral detergent fiber on ash free-basis
A:P: acetate to propionate ratio
ASV: amplicon sequence variant
CH_4_: methane
CO_2_: carbon dioxide
CP: crude protein
DM: dry matter
E: effluent
EMPS: estimated microbial protein synthesis
FL: fermenter liquid
FR: feed residues
FV: *Fucus vesiculosus*
H_2_: hydrogen
LAM: liquid-associated microbes
SAM: solid-associated microbes
OTU: operational taxonomic units
PCoA: principle-coordinate analysis
PCR: polymerase chain reaction
r: correlation coefficient
RF: rumen fluid
RSP: rumen solid phase
TMR: total mixed ration
TPC: total phenolic content
VFA: volatile fatty acids.
