# Supplementary figures and images for: Methane Reduction Potential of Brown Seaweeds and Their Influence on Nutrient Degradation and Microbiota Composition in a Rumen Simulation Technique
Source: Front Microbiol. 2022 Jun 28;13:889618. doi: 10.3389/fmicb.2022.889618 (PMC9273974; doi:10.3389/fmicb.2022.889618)

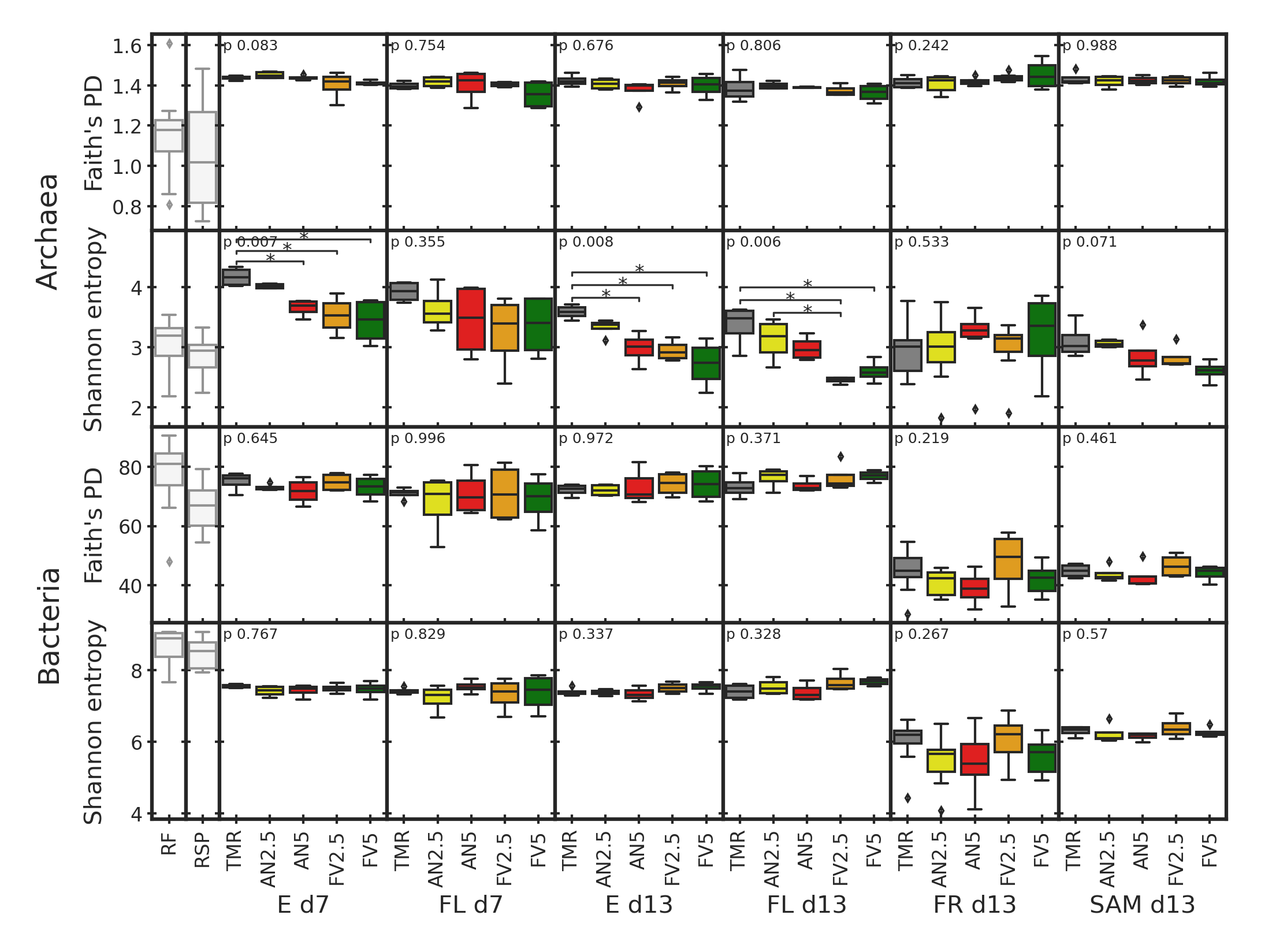

Supplement: Supplementary Figure 1 — Alpha diversity indices by treatment, sample-type and day of sampling. Faith’s phylogenetic diversity and Shannon entropy index are plotted for archaea and bacteria. Each subplots represents one of alpha diversity metrics at a certain day of sampling. Boxplots visualize alpha diversity metrics distribution across all treatments inside of a certain group. Kruskal–Wallis H-test was performed to test the differences between treatments alpha diversity and corresponding P-values are plotted on the top of subplots. Significant P-values denoted as ‘*’ if P ≤ 0.05. [file Image_1.PNG]

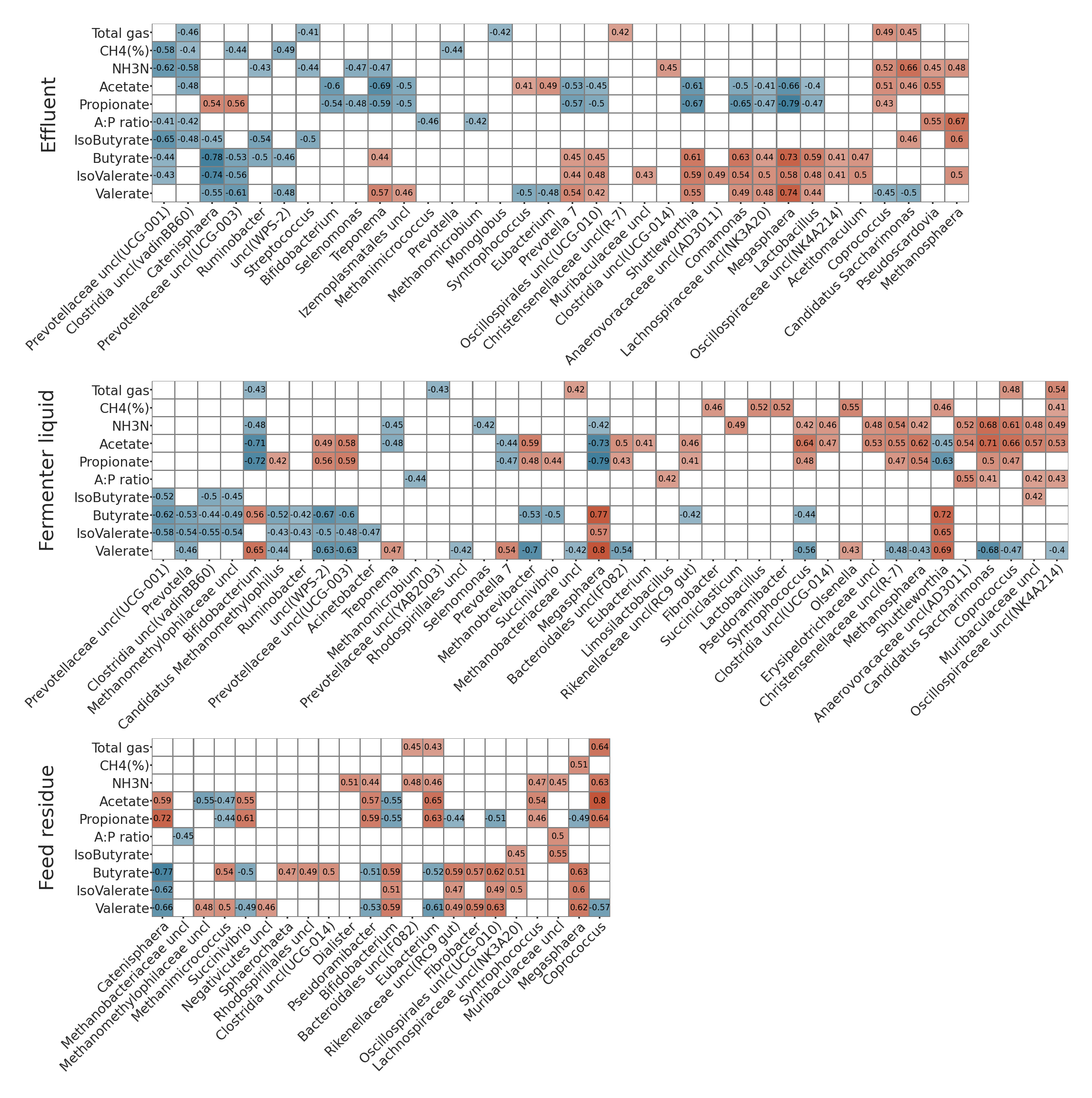

Supplement: Supplementary Figure 2 — SCNIC based correlations of microbial relative abundances among effluent, fermenter liquid and feed residue sample types with total gas production, methane as a percentage of total gas [CH4(%)], ammonium nitrogen in effluent (NH3-N), VFA production and acetate to propionate ratio (A:P ratio). Only correlation coeffitients (r) with absolute values ≥0.3 and adjusted P-values ≤ 0.05 are plotted. Positive correlations are colored in read and negative in blue. [file Image_2.PNG]
